# Structural studies offer a framework for understanding the role of properdin in the alternative pathway and beyond

**DOI:** 10.1111/imr.13129

**Published:** 2022-09-13

**Authors:** Dennis Vestergaard Pedersen, Josefine Lorentzen, Gregers Rom Andersen

**Affiliations:** ^1^ Department of Molecular Biology and Genetics Aarhus University Aarhus C Denmark

**Keywords:** complement, convertase, immune evasion, innate immune system, properdin

## Abstract

Structures of alternative pathway proteins have offered a comprehensive structural basis for understanding the molecular mechanisms governing activation and regulation of the amplification pathway of the complement cascade. Although properdin (FP) is required in vivo to sustain a functional alternative pathway, structural studies have been lagging behind due to the extended structure and polydisperse nature of FP. We review recent progress with respect to structure determination of FP and its proconvertase/convertase complexes. These structures identify in detail regions in C3b, factor B and FP involved in their mutual interactions. Structures of FP oligomers obtained by integrative studies have shed light on how FP activity depends on its oligomerization state. The accumulated structural knowledge allows us to rationalize the effect of point mutations causing FP deficiency. The structural basis for FP inhibition by the tick CirpA proteins is reviewed and the potential of alphafold2 predictions for understanding the interaction of FP with other tick proteins and the NKp46 receptor on host immune cells is discussed. The accumulated structural knowledge forms a comprehensive basis for understanding molecular interactions involving FP, pathological conditions arising from low levels of FP, and the molecular strategies used by ticks to suppress the alternative pathway.

## 1 PROPERDIN IS POSITIVE REGULATOR OF THE ALTERNATIVE PATHWAY

**FIGURE 1:**
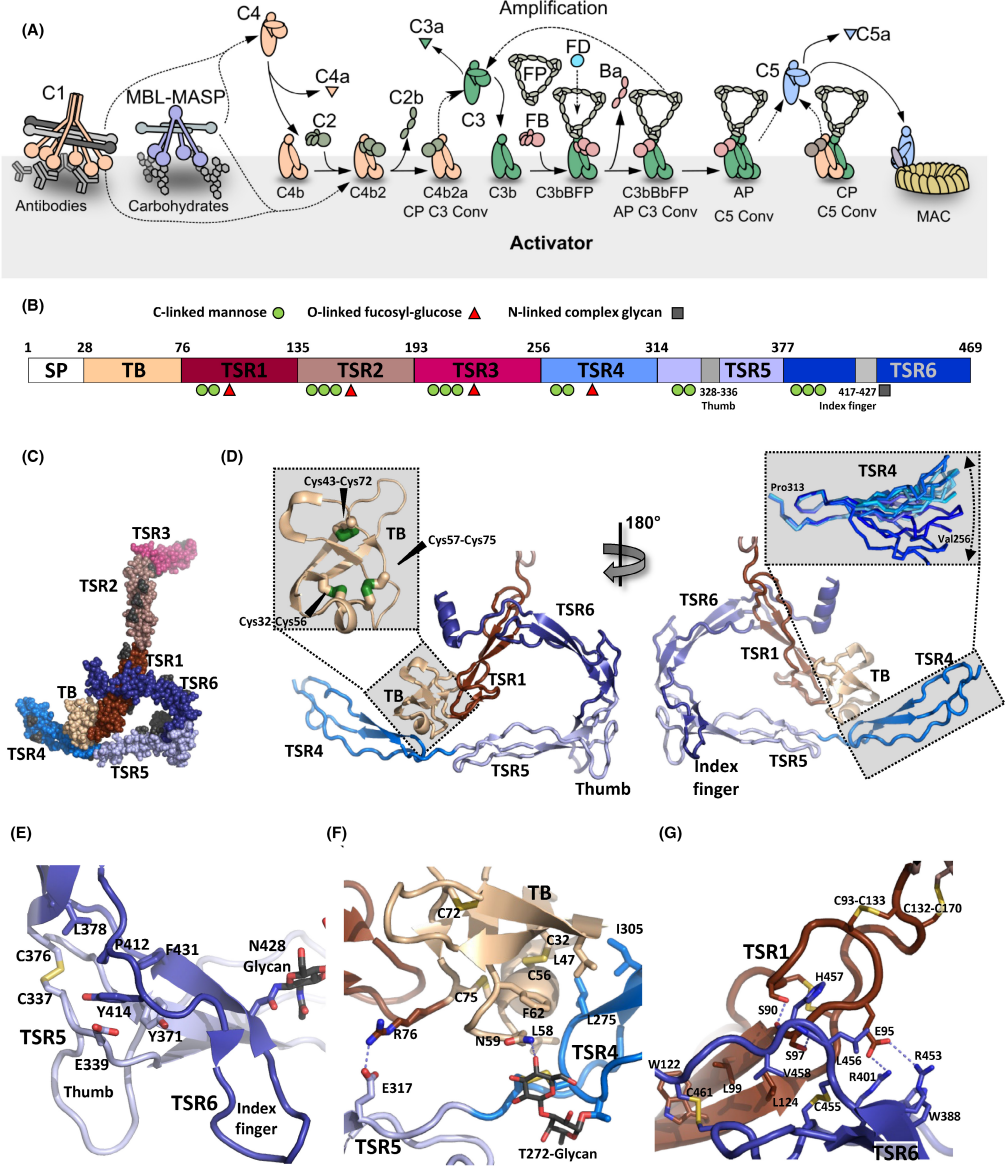
Function and structure of FP. (A) Schematic representation of selected parts of the complement cascade relevant for understanding FP function in the alternative pathway and the downstream terminal pathway. (B) Outline of the FP domain structure with the location of glycans commonly observed in structures of FP marked. The positions of the thumb and index finger loops in TSR5 and TSR6 are also indicated. (C) The structure of the two‐chain monomer FPc with the domains colored as in panel B. This is the only structure encompassing TSR3. (D) The core of FP is formed by the TB domain, TSR1, TSR4, TSR5, and TSR6. The insert to the left shows the disulfide bridge arrangement within the TB domain. The insert to the right visualizes the internal flexibility within TSR4. (E) TSR5 and TSR6 interacts extensively through a hydrophobic interface at the C‐terminal end of TSR5. (F) The TB domain in one FP chain interacts tightly with TSR4 in the second chain through a hydrophobic interface. (G) The second contact point between the two FP chains is formed between TSR1 and TSR6 including its C‐terminal extension.

The complement cascade is activated when pattern recognition molecules recognize pathogens, dying host cells or immune complexes. The proteolytic cascades in complement can initiate through the classical pathway (CP) or the lectin pathway (LP) which leads to assembly of the CP/LP C3 convertase C4b2a. This proteolytic enzyme turns over complement C3 into the anaphylatoxin C3a and the opsonin C3b that may become covalently linked to the surface of the complement activator (Figure 1A). The downstream alternative pathway (AP) provides an amplification loop for the two other pathways.^1^ The C3b initially deposited by the CP C3 convertase can associate with Factor B (FB) and form the AP proconvertase C3bB that upon cleavage by Factor D (FD) becomes the active AP C3 convertase C3bBb.^1^ This initiates a positive feedback loop, which markedly amplifies the complement activation through the CP and LP.^2^, ^3^ FP is central in the AP amplification loop and binds directly to C3b, the C3bB proconvertase, and the C3bBb convertase (Figure 1A). The alternative pathway may also initiate in the fluid by spontaneous hydrolysis of a thioester in C3. The hydrolysis product C3(H2O) functionally resembles C3b and associates with FB^4^, ^5^ and forms the fluid‐phase AP C3 convertase called C3(H2O)Bb.^6^ This fluid phase convertase in principle provides a tonic level of fluid phase C3b that can initiate the AP on cells not presenting complement regulators, but the in vivo relevance of this convertase has been challenged.^7^, ^8^

FP is a positive regulator of the alternative pathway and increases the activity of both the AP C3 and the AP C5 convertase.^9^, ^10^, ^11^ In contrast, FP appears to inhibit the activity of the CP C5 convertase^12^ by interfering with the function of C3b in this convertase. FP is produced predominantly by monocytes, T‐cells, and neutrophils.^13^ The 53 kDa protein is organized in an N‐terminal TGF‐β binding (TB) domain followed by six thrombospondin type I repeats (TSR1‐6) (Figure 1B). The protein is heavily post‐translationally modified and carries one N‐linked glycan, four O‐linked disaccharide glycans and 14–17 C‐mannosylated tryptophan residues in the WxxW motifs present in TSR1‐6.^14^, ^15^, ^16^ In plasma and serum, FP is present as dimers, trimers, and tetramers with a 1:2:1 molar distribution at a concentration of 4–25 μg/ml.^17^, ^18^ The mechanism behind FP stimulation of AP activity has multiple contributions; (a) FP enhances the recruitment of FB to C3b and thereby stimulates proconvertase assembly; (b) FP slows the dissociation of Bb from C3b 5–10 fold; and (c) FP directly competes with factor I (FI) resulting in decreased irreversible degradation of C3b to iC3b.^9^, ^15^, ^19^, ^20^

## 2 THE STRUCTURE OF THE TWO‐CHAIN FP MONOMER

Early negative stain (ns) EM studies revealed that FP oligomers contain compact eye‐shaped vertexes connected by thin connecting structures.^21^, ^22^, ^23^ More detailed structural studies of FP was lagging behind those of all other AP components due to the polydisperse oligomer distribution and the extended architecture of the molecule. A breakthrough was obtained when we inserted a cleavage site for the TEV protease between TSR3 and TSR4.^19^ We could then express oligomeric properdin containing this protease recognition site, and upon TEV cleavage a two‐chain FP monomer was formed that we termed FPc (Figure 1C,D). In this monomer, one chain is formed by the TB domain, TSR1, TSR2 and TSR3 from one FP subunit whereas the another chain contains TSR4, TSR5, and TSR6 from the second FP subunit. These two chains are held together by strong noncovalent interactions.^19^ A simpler strategy was implemented later when we and the Gros group expressed FP and deletion mutants lacking TSR2 and TSR3 as two separate chains with the N‐terminal fragment containing at least TB‐TSR1 and the C‐terminal fragment TSR4‐6. During intracellular maturation and secretion, the two chains assemble correctly to form the mature two‐chain monomeric FP.^16^, ^24^ Importantly, all these two‐chain FP monomers possess the ability to bind to C3b/C3bB/C3bBb, stimulate the assembly of the proconvertase C3bB and retard the spontaneous dissociation of the convertase C3bBb.^15^, ^16^, ^19^

There are now multiple crystal structures of monomeric two‐chain FP monomers that agree in all aspects including post‐translational modifications, disulfide bridge pattern, and quaternary structure (Figure 1C,D, Table 1). Whereas the six TSRs had been predicted since 1988,^25^ it was only with the first structures and accompanying mass spectrometry analysis^15^ it became clear that FP residues 28–76 fold into a TB domain (*left insert* Figure 1D) and not a truncated thrombospondin repeat termed TSR0 as earlier proposed.^26^ The following six TSRs adopt the expected fold with three strands A, B, and C. Strands B and C at the C‐terminal end of each TSR domain pair in an antiparallel two‐stranded β‐sheet. The A strand containing the WxxWxxWxxCxx(S/T)C TSR motif is rippled due to the presence of two residues between each tryptophan. These tryptophan side chains are stacking with arginine side chains from strands B and C as known from other structures containing TSRs.^27^ The Trp‐bound α‐mannosyl residues adopt the ^1^C_4_ chair conformation. The pyranose ring has a preferred orientation wherein the 2‐OH interacts with the tryptophan main chain amide. Furthermore, the endocyclic pyranose oxygen in the mannose in most of these cases accepts a hydrogen bond from an arginine side chain stacking with the tryptophan.^15^, ^16^ It appears that the mannosylations stabilizes the Trp‐Arg stack through hydrogen bonding with protein groups. A stabilizing effect of the mannosylations during folding of TSRs in the endoplasmatic reticulum was recently documented for the netrin receptor UNC‐5.^28^ Assuming this applies to FP as well, it would offer an explanation for the effects for some of the mutations associated with FP deficiencies as discussed below.

**TABLE 1:** Structures of properdin and its complexes. PDB entries are available at the protein data bank, https://www.rcsb.org/. SAXS structures are available from https://www.sasbdb.org/. The atomic model in SASDB59 is obsolete as it was calculated prior to access to the crystal structures.

Crystal structures involving FP				
Entry	Resolution (Å)	Year	Structure	Reference
6RUR	6.0	2019	FPΔ2,3 two‐chain monomer in complex with the SCIN stabilized C3bBb convertase bound to properdin	15, 19
6SEJ	3.5	2019	FPΔ3 two‐chain monomer	^15^
6RV6	3.5	2019	FPΔ3 two‐chain monomer	
6RUS	2.8	2019	FP two‐chain complete monomer	
6S08	2.0	2019	FPΔ2,3 two‐chain monomer	^16^
6S0A	2.5	2019	FPΔ3	
6S0B	2.3	2019	FPΔ2,3 two‐chain monomer in complex with C‐terminal domain of C3	
7B26	3.4	2022	FPΔ2,3 two‐chain monomer in complex with CirpA1	^29^
7NOZ	3.9	2022	FPΔ3 two‐chain monomer in complex with proconvertase C3bB

SAXS structures involving FP				
Entry		Year	Structure	Reference
SASDKA4		2021	FP dimer	^30^
SASDKB4		2021	FP trimer	
SASDKC4		2021	FP tetramer	
SASDB69		2021	FP monomer	
SASDB59		2017	FP two‐chain complete monomer	^19^

Two surfaces in each TSR can be defined: a convex surface containing the Trp‐Arg stack and an opposite concave surface. The only structure that contains the TSR3 domain is that of FPc (Figure 1C) which makes it the obvious reference structure.^15^ The remaining crystal structures lack TSR3 or TSR2‐TSR3 (Table 1).

The overall shape of FPc is dominated by an elongated triangle formed by the TB domain and TSR1 from one FP subunit and TSR4, TSR5, and TSR6 from a second subunit (Figure 1C,D). The N‐terminal end of TSR4 extends as a short arm from this triangle whereas TSR2 and TSR3 protrudes as the second longer arm. In our FPc structure, Pro255 at the C‐terminal end of TSR3 and Val256 at the N‐terminal end of TSR4 are separated by more than 13 nm reflecting that they come from two different FP subunits (Figure 1C). Compared with TSR1‐3 that fold essentially as TSRs in other proteins, the C‐terminal TSRs are unusual. In TSR4, the third tryptophan in the TSR motif is missing which allows internal flexibility as described below.^15^, ^16^ In TSR5, instead of having three residues between the two cysteines in the TSR motif with an O‐linked fucose‐glucose disaccharide as in TSR1‐TSR4, an ordered non‐glycosylated loop is formed by residues 328–336 that protrude at the concave face of TSR5 at its C‐terminal end (Figure 1D). Below we refer to this loop as the TSR5 “thumb” loop, but it is also known as the TSR5 stirrup.^16^ The C‐terminal TSR6 is even more unique and has an unusual large insert of 31 residues between β‐strands B and C. This insert folds back over TSR5 and encompasses a hairpin‐like loop termed the “index finger” loop (or the TSR6 stirrup) that together with the TSR5 thumb loop protrudes at the concave face of TSR5 (Figure 1D). The TSR5‐6 domain interface is unusually large (977 Å^2^) compared with other interfaces between neighboring TSRs and involves a hydrophobic core formed by highly conserved residues (Figure 1E). As a consequence, TSR5‐TSR6 adopts a fixed relative orientation that is strongly bent compared with other pairs of neighboring TSRs in FP.^15^, ^16^

A structural comparison of available FPc and FPΔ3 structures reveal hinges at the TSR1‐TSR2 and TSR4‐TSR5 junctions. At the TSR1‐TSR2 interface a disulfide bridge is formed between Cys132‐Cys170 from TSR1 and TSR2 (Figure 1G), respectively, but this disulfide apparently allows flexibility at the TSR1‐TSR2 junction as the rotation of TSR2 relative to the TB‐TSR1‐5‐6 triangle differ by almost 40° in the known structures. With respect to TSR4, there are only slight differences in the orientation relative to TSR5 in the known structures, but comparison of TSR4 from known structures reveal a surprisingly large internal flexibility between the N‐terminal and the C‐terminal halves of this domain (*right insert* Figure 1D). The N‐terminal half of TSR4 starting with valine 256 can rotate with as much as 60° compared with the C‐terminal half. Since a pretzel‐shaped single chain compact monomeric FP can form as detailed below, hinges must be present also at the TSR2‐TSR3 and TSR3‐TSR4 junctions, otherwise TSR2 and TSR3 would not be able to connect TSR1 and TSR4 in the single chain FP monomer. In summary, the known structures of the two‐chain FP monomers suggest the presence of hinges between TSR1‐2, TSR2‐3, TSR3‐4, and TSR4‐5. In addition, whereas the other TSRs adopt highly similar structures in the known FP structures, TSR4 is unique and has considerable internal flexibility that allows this thrombospondin repeat to adopt a spectrum of conformations.^15^, ^16^

The available structures of FP reveal how extensive non‐covalent interactions formed between TB‐TSR1 from one FP subunit and TSR4‐6 from a second FP subunit support oligomer formation. The TB domain interacts with TSR4 from the second FP subunit while the concave face of TSR1 interacts with a short C‐terminal extension of TSR6 from the second FP subunit (Figure 1F,G). The interface between the two chains is ~1100 Å^2^ with 60% in the TSR1‐TSR6 interface and 40% at the TB‐TSR4 interface, and besides being bigger, the TSR1‐TSR6 interface is also the more hydrophobic. The TB‐TSR4 interface is centered on Leu58, Cys312, and Leu275 with smaller contributions from residues Leu47, Phe62, and Ile305 (Figure 1F). Nearby, a salt bridge between Arg76 and Glu317 links the TB domain with TSR5. The core of the TSR1‐TSR6 interface is formed by the interaction of Pro399 with Leu124, the stacking of the Trp122 with the Cys395‐461 disulfide and hydrogen bonds between Ser97‐Leu456 and Ser90‐His457 (Figure 1G).

## 3 A TSR3 MUTATION FAVORS FORMATION OF A ONE‐CHAIN PRETZEL‐SHAPED FP MONOMER

**FIGURE 2:**
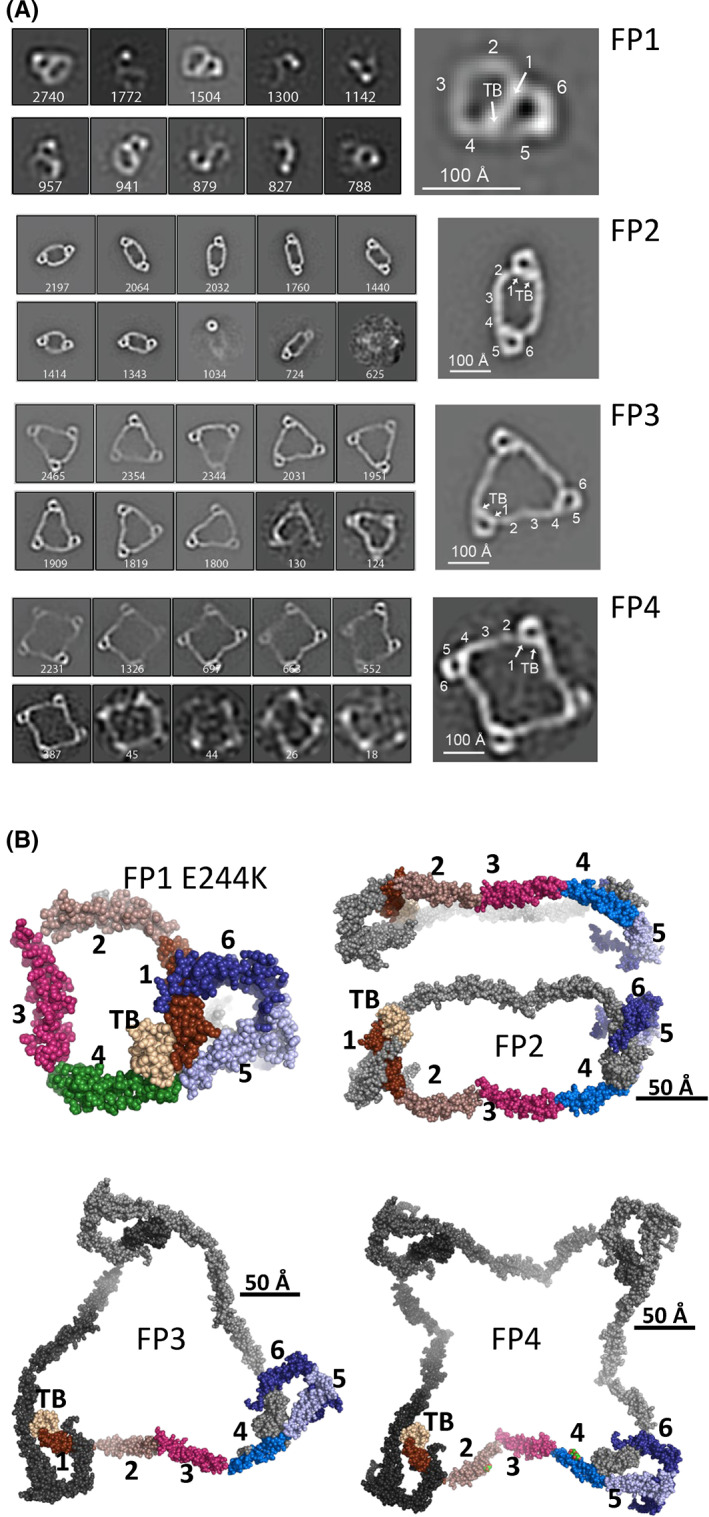
The structure of FP monomer and oligomers. (A) To the left are displayed the most populated 2D classes from negative stain EM analysis. To the right, a magnified view of one representative 2D class with the TB domain and the TSRs assigned. (B) A single representative model obtained by rigid body refinement against SAXS data for the four FP states investigated. For FP2, two orientations are displayed to demonstrate the curved nature of the FP dimer. For each of the FP oligomers, the domains in one subunit are colored as in Figure 1B. Figure adapted from reference.^30^

FP normally circulates as a mixture of dimers, trimers, and tetramers in plasma, but we discovered that mutation of FP glutamate 244 to lysine caused the resulting recombinant FP E244K to be secreted from mammalian cells almost exclusively as a monomer.^19^ Interestingly, during preparation of recombinant non‐mutated FP, it is also possible to purify minute amounts of the wild‐type monomer that we term FP1 in which the oligomer interfaces described above are formed within a single FP molecule.^30^ In 2D classes obtained by negative stain EM, FP1 appeared as a flat pretzel‐shaped molecule with apparent overall projected dimensions of 95 × 115 Å containing a small and a large ring‐shaped structure (Figure 2A). The small ring of the FP1 molecule corresponds to the FP triangle formed by the TB domain, TSR1, TSR5, and TSR6,^15^, ^16^ whereas TSR2, TSR3, and TSR4 together with the TB domain and TSR1 delineate the large ring.

Starting from our crystal structure of the two‐chain monomer FPc, we constructed a pseudo‐atomic model in accordance with the FP1 2D classes. We used this model to conduct rigid body refinement against small angle X‐ray scattering (SAXS) data obtained for FP1 carrying the E244K mutation.^19^ The resulting SAXS‐based models (Figure 2B) strongly resembled the EM 2D classes obtained for the wildtype FP1 monomer.^30^ Hence, the E244K in TSR3 mutation does not appear to cause overall changes in the structure but must favor intra‐subunit monomer formation over inter‐subunit oligomer formation during maturation and secretion. Molecular dynamics simulations of FP1 suggested a certain rotational freedom of the TSR3 domain and also recapitulated the internal flexibility within TSR4 suggested by crystal structures (Figure 1D).^30^

## 4 THE STRUCTURE OF FP OLIGOMERS

In 2004, Perkins and co‐workers investigated the solution structure of FP dimers and trimers with SAXS and analytical ultracentrifugation^26^ but generation of atomic models fitting these data were at that time complicated by the lack of information regarding the quaternary structure of FP oligomers and the unknown fold of the N‐terminal region as a TB domain. Recently, we interpreted data obtained for FP dimers (FP2), trimers (FP3), and tetramers (FP4) with SAXS and negative stain EM through an integrative approach that took into account the available crystal structures of FP (Table 1).^30^ Comparison with the crystal structures enabled us to identify all the domains in FP2, FP3, and FP4 in EM 2D classes for all three oligomers (Figure 2A). Well‐defined 2D classes require that the individual molecules for which micrographs are averaged adopt a similar conformation, otherwise, the molecule will appear blurred in 2D classes. Clear 2D classes were obtained for especially FP dimers and trimers, but even for the FP tetramer 2D classes, the individual TSRs could easily be recognized by comparison with the crystal structures. The observed rigidity of the three different FP oligomers that enabled well‐defined EM 2D classes was not anticipated in the light of the hinges apparent from the comparison of available two‐chain monomer structures (Table 1) and opposes the common perception of FP oligomers as being flexible molecules.

In negative stain EM, the analyzed molecules associate with the grid and staining with a heavy metal solution is performed. This procedure potentially could have trapped the FP oligomers in conformations that are not in vivo relevant. Furthermore, the 2D classes of all the investigated FP species only presented views of the molecule in the preferred orientation where the two major axes of the molecules are in the plane of the image (Figure 2A). Hence, the EM micrographs could not be used to obtain a 3D reconstruction of the FP monomer and oligomers. In an orthogonal approach, we therefore took advantage of size exclusion chromatography‐SAXS analysis where the data for carefully separated FP oligomers were collected under physiologically relevant buffer conditions.^30^ It is noteworthy that both the SAXS scattering curves and derived model‐independent pair‐distance distributions calculated for the oligomers exhibit characteristic bumps that are in agreement with that the FP oligomers are rigid and adopt a well‐defined average conformation.^30^ If substantial conformational freedom had been present in the oligomers, this would smear out the SAXS data. Using rigid body refinement of oligomer models against the corresponding SAXS data, we obtained models of the three different oligomers. The SAXS model of FP2 appeared more curved than the extended conformations observed in EM 2D classes of FP2 (Figure 2A,B), which could be due to an effect of the EM grid or the stain. In contrast, models of FP3 and FP4 obtained by rigid body modeling agreed rather well with their appearance in the EM 2D classes. Our SAXS analysis of FP oligomers also suggested that the FP3 and FP4 oligomers are close to being symmetric whereas the average FP2 conformation may be non‐symmetric. A non‐symmetric structure of the dimer may appear counterintuitive but is well established for the homodimeric IgE antibody that is also rigid at the resolution of negative stain EM.^31^ Our ability to explain solution scattering data for all three oligomers with single models further strengthens the idea that FP oligomers adopt a limited number of overall similar conformations in solution. Intriguingly, we observed more compact conformations of the oligomers in the presence of the TSR4‐specific nanobody hFPNb1 in negative stain EM,^30^ but the physiological relevance of this is uncertain as the nanobody has no influence on the ability of FP to stimulate AP activity.^15^

## 5 PROPERDIN RECOGNIZES PRIMARILY C3b IN THE CONVERTASE

**FIGURE 3:**
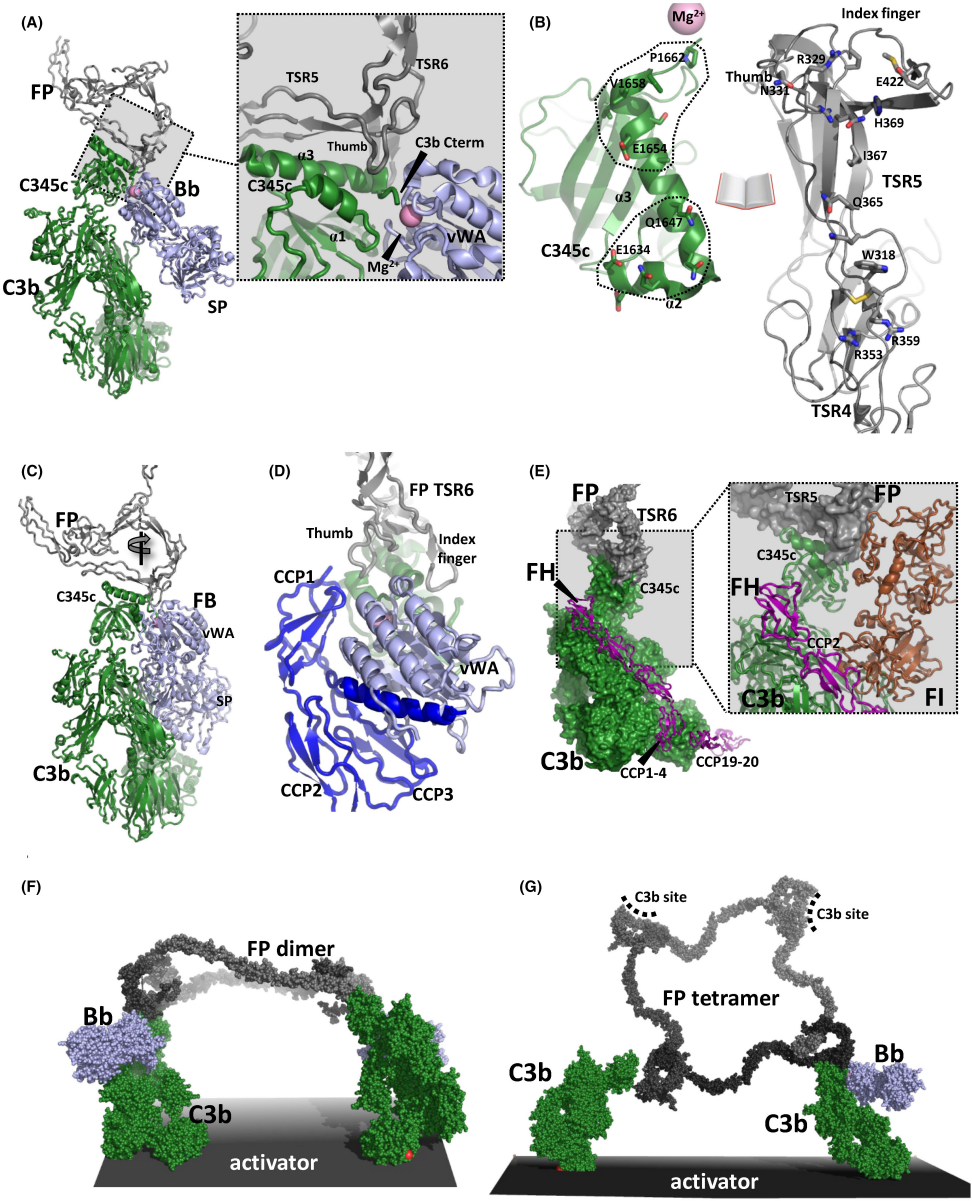
Structure of FP in the (pro)convertase complex, competition with FI and implications of oligomer structure for activator bound FP oligomers. (A) Structure of C3bBb‐FP complex from the entry 6RUR.^15^ The SCIN protein used for locking the complex is not shown. The Mg^2+^ ion in the Bb MIDAS coordinated by the C‐terminal carboxy group in C3b is depicted as a pink sphere. (B) An open book representation of the interface with contacting residues in C3b C345c and FP shown as sticks. Notice that both the thumb and index finger in FP are in contact with the C345c domain. To the left, the two different binding patches are outlined. The top patch at the C‐terminal end of the α3‐helix is the more nonpolar while the lower patch is rather polar. (C) Structure of the proconvertase C3bB‐FP complex in entry 7NOZ. The C3b β‐chain is oriented as in panel A. A bifunctional nanobody used to stabilize the complex is not shown. (D) When bound to the proconvertase, the FP thumb loop inserts between the FB CCP1 domain and the C3b C345c domain. The Ba moiety within FB is colored blue and the vWA domain lightblue. The FB serine protease domain is not shown. (E) Modelled structure of the ternary complex between FH, C3b and FP obtained by combining entries 7NOZ and 5O32. In accordance with experiments, the model predicts that FP and FH can bind simultaneously. In the insert to the right, FI has been added to the model. A small but significant overlap occurs between FP and the N‐terminal part of FI in agreement with slower FI degradation in the presence of FP.^15^ (F) Modelled structure a FP dimer bound to two C3bBb complexes on a planar activator surface. Due to the curved structure of the dimer, simultaneous binding through two C3b binding sites appears possible. (G) As in panel F, but with a FP tetramer. On a planar activator, binding to more than two C3b sites is unlikely to take place for FP trimers and tetramers.

The AP C3 convertase is unstable and dissociates irreversibly into C3b and Bb and is therefore not an optimal sample for structural studies. In 2009, Roijakkers and colleagues demonstrated that a small protein from Staphylococcus aureus called SCIN traps C3bBb in stable complex where the convertase is dimerized and inactive.^32^ As a breakthrough in our understanding of the alternative pathway, they were able to determine the structure of the inhibited SCIN stabilized C3bBb AP C3 convertase.^32^ We also took advantage of SCIN to investigate how FP interacts with C3b and Bb and determined the structure of the SCIN stabilized C3bBb‐FPc at a resolution of 6 Å.^15^, ^19^ The C3b‐FP interface we observed at low resolution in the SCIN stabilized C3bBb‐FP complex and the crucial role of the FP thumb and index finger loops were confirmed by the crystal structure of the minimal complex between FPΔ2,3 and the C3 C345c domain.^16^ Minor differences in the mode of C3b‐FP interactions in these two structures may be caused by the absence of Bb in this minimal complex (Figure 3A,B). These two structures revealed that the FP binding site on the AP C3 convertase is dominated by C3b residues located in two separate patches contained within C3b residues Glu1634‐Phe1659 in two α‐helices α2 and α3 within the C‐terminal C345c domain of C3b. Furthermore, a few additional putative contacts appear to be formed between FP and the Bb von Willebrand factor type A (vWA) domain.^15^, ^19^ The C3bBb subunits are recognized by the concave face of FP TSR5 including the “thumb” loop together with the TSR6 “index finger” loop (Figure 3A,B). The major FP‐C3b contact occurs close to Bb and presumably involves a mixture of polar and non‐polar interactions. A saddle‐shaped surface formed by TSR5 including the thumb loop and the TSR6 index finger surrounds the surface exposed C3b side chains of Val1657, Val1658, and Phe1659 (Figure 3B). The coordination of the Mg^2+^ ion in the Bb metal ion dependent adhesion site (MIDAS) by the C‐terminus carboxylate from C3b is crucial for the C3b‐Bb interaction. In our structure of the FP‐convertase complex, the three C‐terminal residues of C3b Cys1661‐Asn1663 are sandwiched between the FP TSR5 thumb loop and Bb (*insert* Figure 3A). This stabilization may be an important component in the mechanism whereby FP slows down the dissociation of Bb from C3b. The second major FP‐C3b contact appears to involve mainly electrostatic and other polar contacts. This interface contains FP residues organized around the side chain of Trp318 including Arg353 and Arg359 and polar C3b residues located between Glu1634 and Gln1647 (Figure 3B). These C3b residues include multiple negatively charged side chains, and the electrostatic potentials of the interacting C3b and FP surfaces suggest that ionic interactions play a major role at this part of the interface.^15^

To understand how FP promotes assembly of the proconvertase C3bB, we very recently determined the structure of the C3bB‐FP complex by linking FP and FB with a bifunctional nanobody.^33^ Here, FP binds to C3b in the proconvertase essentially as in the downstream activated C3bBb‐FP convertase complex (*compare* Figure 3A,C). As observed in the C3bBb‐FP complex, the TSR6 index finger loop and the TSR5 thumb loop are strongly involved in FP‐FB interactions. The TSR5 thumb loop is located in a pocket formed by the FB vWA domain, the first FB CCP domain and the small α‐helix 1 in the C3b C345c domain. Additional FP‐FB contacts appear to take place between FB and the FP TSR6 index finger loop (Figure 3D).

Cofactor‐dependent degradation of C3b by FI is an important regulatory mechanism of the alternative pathway on host tissue. Factor H (FH) is a soluble cofactor that through interaction with surface glycan allows C3b degradation by FI (reviewed in ^34^). Early studies demonstrated that with FP present, FI interaction with erythrocyte bound C3b was inhibited suggesting that FP inhibits C3b degradation by competing with FI.^35^ Our structure of the C3bBb‐FP complex confirmed the overlap between the binding sites for the FI FIM domain^36^ and FP on the C3b C345c domain (*insert in* Figure 3E). Using immobilized mini‐FH,^37^ we also confirmed that C3b can bind mini‐FH and dimeric FP simultaneously^15^ confirming classic competition experiments that revealed distinct binding sites for FH and FP.^38^ In a system of purified components, we showed that the presence of FP oligomers significantly delayed FH‐assisted FI degradation of immobilized C3b to iC3b in agreement with a direct competition between of FP and FI.^15^

## 6 C3 CONVERTASE AND FP OLIGOMERS

The activity of FP oligomers in complement‐dependent erythrocyte lysis increases with oligomer size,^18^ and the two‐chain monomer FPc and the single‐chain monomer FP1 E244K are also much less active in convertase stabilization on erythrocytes and bactericidal activity compared with oligomeric FP.^19^ An important question concerning FP function is whether the positive correlation between stimulation of AP activity and oligomer size is caused by simultaneous binding to multiple C3b molecules and convertases deposited on an activator. In FPn, there are n binding sites for C3b each located at the concave face of TSR5. Our structures of the C3bBb‐FP and C3bB‐FP complexes (Figure 3A–D) demonstrated that C3b binds with its major axis roughly parallel to the plane of the FP triangle formed by the TB domain, TSR1, TSR5, and TSR6.^15^ Combination of the structure for the C3bBb‐FP complex in Figure 3A with the structures of the three different FP oligomers in Figure 2B allows us to investigate whether oligomers may simultaneously bind more than one C3 convertase on a planar C3b opsonized activator. In this case, the major axis of deposited C3b molecules are roughly parallel (Figure 3F). Since FP oligomers become increasingly planar as their size increases, we proposed that simultaneous binding to more than one C3b/convertase molecule deposited on such a planar activator becomes increasingly unlikely as the oligomer stoichiometry increases.^30^ The higher biological activity of the larger oligomers is therefore likely to be caused by an increased local concentration of C3b binding sites favoring FP rebinding after dissociation rather than simultaneously multivalent interaction between the FP oligomer and the C3b opsonized activator.^30^ However, if the activator is non‐planar and the major axis of C3b can take many different orientations, multivalent FP‐C3b interaction may occur. Interestingly, the high activity of the larger FP oligomers can only be partially restored by linking FPc monomers together with a multivalent FP‐specific nanobody suggesting that the well‐defined extended structure of FP oligomers forms an important contribution to the biological activity of FP oligomers.^30^

## 7 FP AND THE C5 CONVERTASE

C5 convertases are formed from the alternative pathway C3 convertase C3bBb and the homologous classical pathway C3 convertase C4b2a (Figure 1A) at a high density of C3b on the activator.^39^, ^40^ The C5 convertases initiate the terminal pathway where cleavage of complement C5 results in release of the potent proinflammatory anaphylatoxin C5a that activates a range of cell types through binding to the C5aR1 receptor.^41^ Furthermore, C5b is generated and nucleates the assembly of the membrane attack complex that may lyse pathogens but also host cells with defects in complement regulation as exemplified by the lysis of red blood cells in paroxysmal nocturnal hemoglobinuria.^42^ The exact composition and structure of the C5 convertases are not clear, it remains to be settled whether there is an additional C3b molecule directly associated with both C5 convertases or the role of the high C3b density is to recruit and prime C5 for cleavage by a C3 convertase (reviewed in ^43^). Since FP promotes the required high C3b density through its stimulation of the AP C3 convertase, it is not surprising that FP deficiency or blockade inhibits the terminal pathway.^44^ At the high C3b density required for formation of C5 convertases, oligomeric FP must bind avidly. The FP stabilized alternative pathway C3‐ and the C5‐convertases decay by Bb dissociation with similar kinetics^10^ suggesting that FP binds to C3bBb within the alternative pathway C5 convertase as in the C3 convertase. Intriguingly, a recent study showed that addition of FP after assembly of AP convertases in FP deficient serum and removal of C3 not bound to the erythrocytes was required for the activity of the C5 convertase in a hemolysis assay.^11^ This possibly mirrors that FP prevented spontaneous Bb dissociation from the AP C5 convertase during the assay. In contrast, FP binding to C3b has been reported to inhibit the classical pathway C5 convertase activity without interfering with CP C3 convertase activity^12^ indicating that the C3b C345c domain presenting the FP binding site have a function in the CP C5 convertase activity. This idea is supported by the partial antagonistic effect on the CP C5 convertase of a C3‐specific nanobody that shares binding sites with FP on the C‐terminal domain of C3/C3b.^45^ Since none of the C5 convertases have been reconstituted in a soluble format, more detailed structural insight on the function of FP in relation to the C5 convertases may require cryo‐EM tomography analysis of vesicles or cells onto which the AP C5 convertase have been formed.^46^ However, considering the similar stabilization of the AP C3‐ and C5‐convertases by FP we predict that the contacts it forms with Bb and the Bb‐associated C3b molecule in a C5 convertase will overall be conserved between the AP C3‐ and C5‐convertases. In addition, it has been suggested that FP by limiting the lateral diffusion of C3b deposited on a cell may stimulate the activity of the AP C5 convertase.^11^ Further functional and structural studies are required to fully understand the contribution of FP to the activity of both C5 convertases.

## 8 A STRUCTURE‐BASED UNDERSTANDING OF PROPERDIN DEFICIENCIES

**FIGURE 4:**
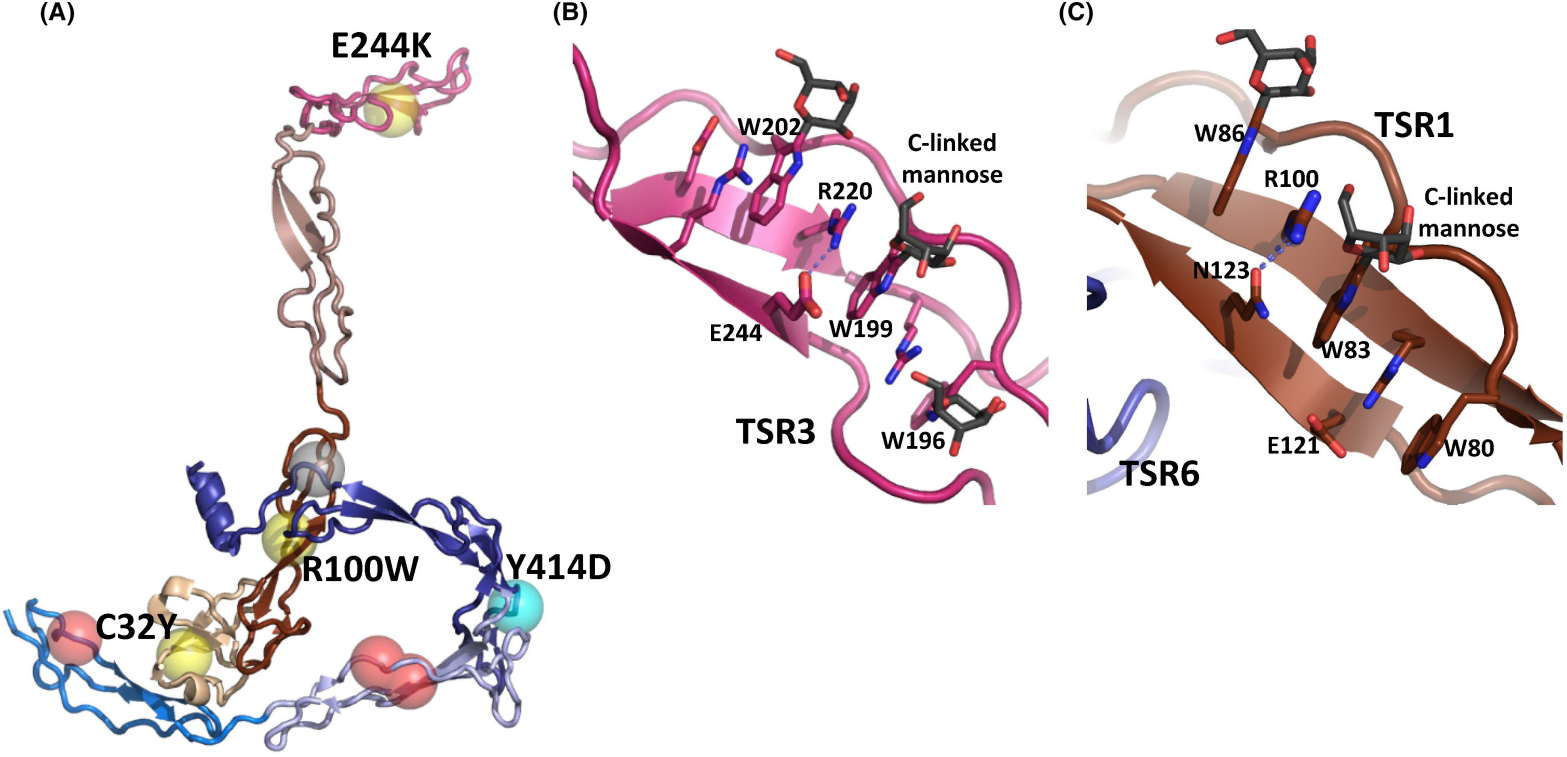
Structural aspects of point mutations associated with FP deficiencies. (A) Overview of the FP mutations causing type I (red spheres), type II (yellow spheres), type III (cyan sphere) deficiency and the uncharacterized L456V mutation (grey sphere). The environments of Cys32 and Tyr414 are presented in Figure 1E,F, respectively. (B) Magnified view of the environment around residue 244 in TSR3. The glutamate engages in an electrostatic interaction with Arg220 that is part of the tryptophan‐arginine stack in TSR3. All three tryptophans in this stack are covalently linked to a mannose. (C) Environment of Arg100, where mutation to tryptophan gives type II deficiency. Substitution with the significantly larger tryptophan side chain is likely to perturb the Trp‐Arg stack in TSR1. Only the second and the third tryptophan side chain are mannosylated in TSR1.

FP deficiency (PD) is an X‐linked disorder that can be divided into three subtypes: type‐I (complete lack of FP), type II (1%‐10% of normal plasma FP level), and type III (normal plasma level but dysfunctional FP). Individuals subject to any type of PD have significantly reduced activity of the alternative pathway, impaired bactericidal activity and susceptibility to *Neisseria* infections and sepsis.^47^ The available crystal structures involving FP offer detailed explanations for why a particular mutation giving rise to FP deficiency causes lack of AP activity (Table 2 and Figure 4). In type I deficiencies, FP is not secreted, which is most likely due to degradation of truncated or misfolded FP under the intracellular quality control occurring during maturation and secretion. Mutations G298V, W321S/G, and R346C cause type I deficiency.^48^, ^49^, ^50^ The presence of a valine side chain instead of Gly298 may prevent insertion of Trp260 in the Trp/Arg stack of TSR4 possibly leading to misfolding of TSR4 and potentially also a decreased stability of the TB‐TSR4 interaction involved in oligomer stabilization. Disruption of the tryptophan‐arginine stack in TSR5 may well be a consequence of the Trp321Ser/Gly as well as the mutation Arg346Cys.

**TABLE 2:** Known mutations in human FP leading to deficiency.

Deficiency	Mutation	Domain	Origin	Reference
Type‐I	Arg79STOP	TSR1	Switzerland, The Netherlands	48, 49, 51
Type‐I	Arg166STOP	TSR2	Sweden	52, 53
Type‐I	Gln187STOP	TSR2	Israel	^50^
Type‐I	Ser206STOP	TSR3	The Netherlands	48, 49
Type‐I	Cys224del. + frameshift, STOP	TSR3	Sweden	^54^
Type‐I	GlyPro262del. + frameshift	TSR4	The Netherlands	48, 49
Type‐I	Gly298Val	TSR4	The Netherlands	48, 49
Type‐I	Trp321Gly	TSR5	South Africa, The Netherlands	48, 49, 54
Type‐I	Trp321Ser	TSR5	The Netherlands	48, 49
Type‐I	Arg346Cys	TSR5	Israel	^50^
Type‐I	Trp388STOP	TSR6	Finland	^55^
Type‐II	Cys32Tyr	TB	France	^15^
Type‐II	Arg100Trp	TSR1	Sweden	53, 56
Type‐II	Glu244Lys	TSR3	France	^19^
Type‐II	Gln343Arg	TSR5	Denmark	^57^
Unknown	Leu456Val	TSR6	Korea	^58^
Type‐III	Tyr414Asp	TSR6	The Netherlands	^59^

In type II deficiencies, the FP serum level is unusually low and the distribution of FP oligomers is abnormal. We recently described a novel type II FP deficiency where Cys32 in the TB domain is mutated to tyrosine.^15^ This disrupts a disulfide bridge in the TB domain (*left insert* Figure 1D) and is likely to compromise the nearby TB‐TSR4 domain interface. Accordingly, only very low levels of recombinant FP C32Y could be expressed in mammalian cell culture compared with the wildtype FP. A young childbearing carrying this mutation had less than 10% AP activity and died due to *Neisseria menigitidis* infection.^60^

As alluded to already, we also described a type II deficiency caused by mutation of glutamate 244 to lysine in TSR3, which results in absence of AP activity. Recombinant FP E244K is secreted in cell culture almost exclusively as a monomer with a compact structure^19^ that is likely to be shared with the wildtype but rare FP1 single‐chain monomer (Figure 2). In our crystal structure of FPc, Glu244 interacts electrostatically with Arg220 (Figure 4B). Based on additional mutants targeting position 244, we proposed that the mutation of 244 to a lysine destabilizes Arg220 and thereby presumably the Trp/Arg stack in TSR3 containing Arg220 (Figure 4B). This most likely translates into a compromised stability of TSR3 and a high probability of intracellular degradation explaining the low abundance of FP in the plasma of E244K carriers.^19^ The failure of FP E244K oligomer formation is more difficult to rationalize but could be a consequence of a low concentration of this FP variant at the stage in intracellular maturation where oligomerization occurs.

The mutation of arginine 100 to tryptophan represents a third example of type II deficiency. As for the E244K mutation, a tryptophan at residue 100 is also likely to disturb the TSR1 Trp/Arg stack (Figure 4C) explaining why the low serum level and an altered oligomer distribution is observed.^57^ Mutation of glutamine 343 to arginine also results in type II deficiency with a shift in the oligomer distribution towards dimers.^57^ This glutamine is part of the C3b binding site and our binding studies showed that its mutation to arginine significantly decreases the FP affinity for C3b.^15^ With respect to type III deficiency, mutation of tyrosine 414 to aspartate gives rise to normal serum FP level and oligomer distribution but the activity of the alternative pathway is compromised. The FP Y414D variant binds weakly to immobilized C3bB and C3bBb^59^ and to C3b itself.^15^ Our structures of FP and its complex with convertase and proconvertase rationalize these results. The mutation of tyrosine 414 to aspartate is likely to lead to repulsion with the neighboring glutamate 339 (Figure 1E), change the conformation of the TSR5 index finger loop and possibly also the thumb loop in TSR6. This could impact the interaction of both loops with FB in the proconvertase complex^33^ and Bb in the convertase complex.^15^ Recently, the combination of heterozygous mutations in IL10RA with the hemizygous FP mutation of leucine 456 to valine was reported in a case of infantile‐onset inflammatory bowel disease.^58^ The FP plasma level and oligomerization state was not reported, and inspection of the available FP structures does not argue for a severe impact of this mutation on the FP structure. However, the residue is almost strictly conserved in mammalian FP, and its mutation could interfere with formation of the disulfide bridge Cys391‐Cys455 (Figure 1G). Further biochemical characterization of this FP variant will be required to judge its functional impact.

## 9 STRUCTURAL BASIS FOR COMPLEMENT EVASION THROUGH INHIBITION OF FP

**FIGURE 5:**
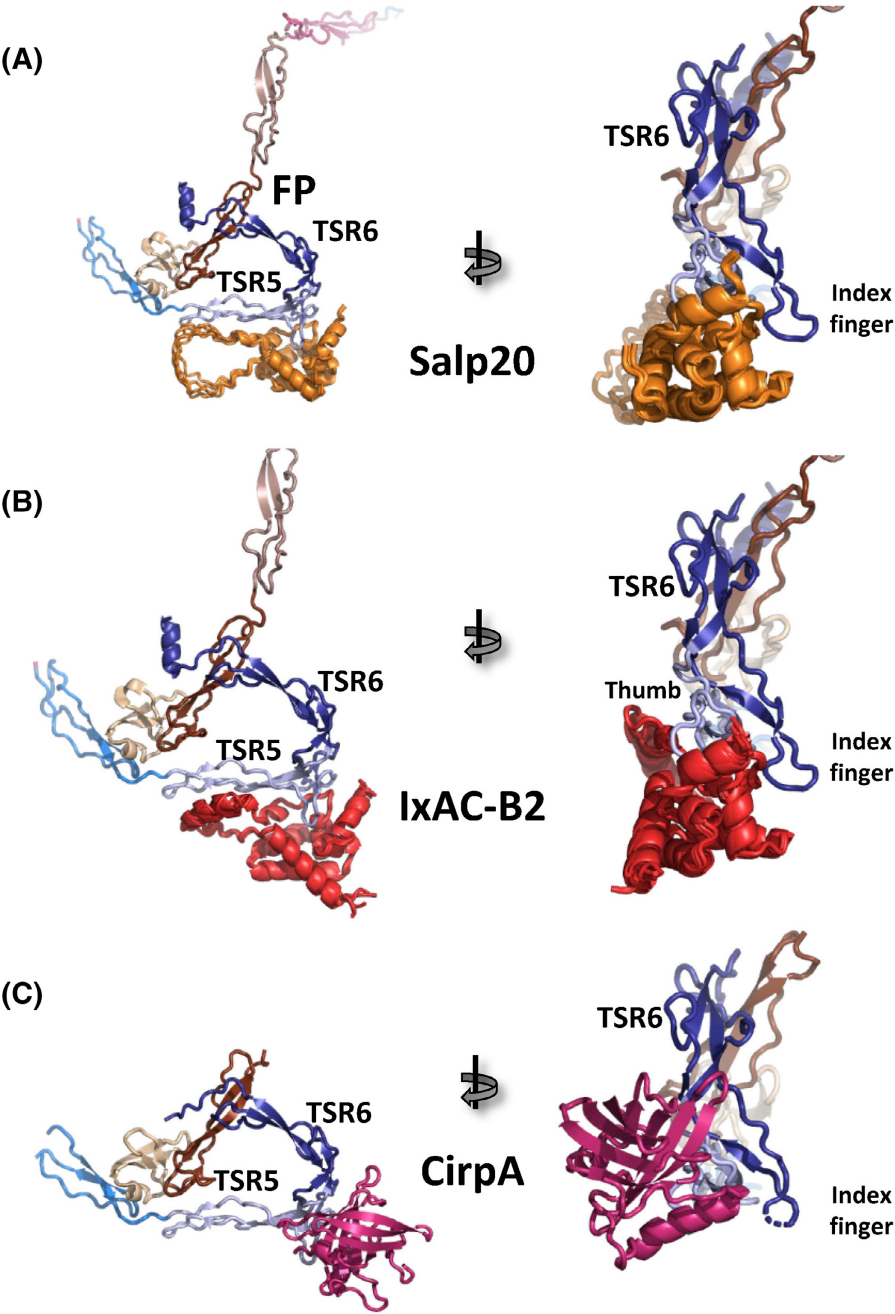
Tick complement inhibitors target the FP thumb loop. (A) Five models of the FP‐Salp20 complex were prepared with alphafold2 and superimposed on the structure of FPc. The resulting Salp2 models (orange) all insert a helical bundle between the TSR5 thumb and the TSR6 index finger but in addition, a helix and a long loop interact directly with the concave face of TSR5 holding the C3b binding site. The FP sequence was used as a single chain. The alphafold2 predicted model was superimposed onto TSR5 in the pdb entry 6RUS to create a combined model of the five Salp20 molecules together with the experimental FPc structure for presentation. (B) As in panel A, but with predicted models of IxAC‐B2‐FP complexes instead of Salp20. The two tick inhibitors are members of the same protein family and alphafold2 predicts them to be highly homologous structurally and to interact with FP through overall similar binding modes. (C) The experimental structure of the CirpA‐FP complex. Like Salp20 and IxCA‐B2, CirpA interacts tightly with the TSR5 thumb loop but only indirectly affects the conformation of the TSR6 index finger. Furthermore, CirpA does not interact with the concave face of TSR5 containing the C3b binding site.

Many pathogens including bacteria, virus, ticks, and yeast evade the innate immune response by surface‐expressed and secreted proteins that interfere with the normal function of complement proteins or degrade complement proteins (reviewed in ^61^). Prior studies characterized the interaction of FP with the Salp20 protein and multiple homologous IxAC protein from the hard ticks Ixodes scapularis and Ixodes ricinus. When bound to these tick proteins, FP fails to stabilize the AP C3 convertase.^62^, ^63^ However, the binding site of Salp20 and homologs tick proteins on FP is unknown. Since these proteins are present in multiple species of hard ticks and inhibit the alternative pathway in mammals broadly,^62^ we investigated whether the modeling procedure alphafold2^64^ that takes into account sequence co‐evolution could predict the structure of the FP‐Salp20 complex. Interestingly, the predicted structure of the complex is in perfect agreement with the ability of Salp20 and other homologous tick proteins to interfere with FP function in the alternative pathway (Figure 5A). Salp20 is predicted by alphafold2 to fold into seven α‐helices that pack tightly into a globular unit without close structural homologs in the protein data bank. According to the model, this globular unit interacts with both the TSR5 thumb and the TSR6 index finger in FP (Figure 5A). Furthermore, a large extended region located between helices 6 and 7 in Salp20 appears to interact mainly though electrostatic interactions with lysine and arginine side chains located at the concave face of FP TSR5 that also houses the C3b binding site (Figure 5A). Prediction of the FP complex with the IxAC‐B2 protein from Ixodes ricinus (a homologue of Salp20) results in a very similar complex, which adds further credibility to both alphafold2 predictions (Figure 5B). However, it should be kept in mind that alphafold2 predictions cannot take into account the mannosylations on TSRs tryptophans, and interactions involving these may therefore not be well predicted. Alphafold2 predicted structures must therefore be carefully verified experimentally, but it is striking that they, in an unbiased manner, offer a plausible explanation of the known functional properties of Salp20 and its homologs.

Recently, Braunger et al. described the CirpA family of tick inhibitors that are ~200 residue proteins also binding directly to FP.^29^ Members of the CirpA family adopt a lipocalin fold that is very distinct from the α‐helical fold predicted for Salp20 and the IxAC proteins (Figure 5). Mechanistically, the CirpA proteins were demonstrated to interfere with FP binding to C3b. The crystal structure of the FP complex with the Cirp1A protein revealed a tight recognition of the TSR5 thumb loop by the tick inhibitor (Figure 5C) that clearly will prevent interaction of FP with both Bb in the AP C3 convertase and FB in the proconvertase according to our structures.^15^, ^19^, ^33^ Presumably, the remaining CirpA family members recognize FP in a very similar manner. In contrast, the FP‐Cirp1A structure does not answer directly how the CirpA proteins interfere with C3b binding since there is no overlap between binding sites for Cirp1A and C3b on FP. But the TSR6 index finger loop is disordered in the FP‐Cirp1A structure^29^ while it is ordered in the structures of the SCIN stabilized convertase C3bBb‐FP,^15^, ^19^ the proconvertase C3bB‐FP^33^ and the minimal complex of the C3 C345c domain and FP.^16^ Hence, a plausible explanation for the inhibition of the FP‐C3b interaction is that binding of CirpA proteins induces a disordered conformation of the TSR6 index finger loop that prevents stable interaction of FP with C3b.

## 10 NON‐CANONICAL INTERACTION PARTNERS

In contrast to the convertase‐related functions, the role of FP as a C3b independent pattern recognition molecule is still up for debate. As reviewed in details in,^65^, ^66^ FP has been proposed to bind directly to pathogens, activated platelets and apoptotic/necrotic host cells and thereby recruit fluid phase C3b or the C3 tick‐over product C3(H_2_O) and support initiation of the alternative pathway amplification on the FP binding pattern. C3b independent recruitment of FP has been questioned,^67^ but in a C3 knockout mouse FP was still deposited at injured glomeruli in vivo although the responsible FP binding pattern was not identified.^68^ Molecular patterns suggested to be recognized by FP include LPS, negatively charged host glycan, zymosan, acetylated LDL, and multiple proteins. Very recently FP4, but not FP3 and FP2, was shown to act as a C3b independent pattern recognition molecule on bacteria by binding soluble collectin‐12 suggesting a specialized function of FP4 as a pattern recognition molecule.^69^ Our structure of the two‐chain FP monomer FPc hints at how FP mediates pattern recognition. Negatively charged sulfated groups on host glycans may form electrostatic interactions with the C‐terminal end of TSR3, where two sulfate ions are firmly bound to an arginine in our crystal structure of FPc.^15^ In addition, a crystal packing interaction involving two TSR2 domains in this structure illustrates how the TSR‐associated mannosylations may form hydrogen bonds with carbohydrate groups on host glycan or glycosylated proteins.^15^

Interestingly, our structures also predict that empty C3b/convertase binding sites in the FP3 and FP4 oligomers point in a direction opposite to the occupied site(s) due to their rather flat structures (Figure 3G). Such unoccupied C3b binding sites in FP3 and FP4 may protrude more than 300 Å from the thioester‐activator linkage of the C3b to which a monovalent C3b‐FP interaction occurs. Empty binding sites presented 30 nm from the C3b opsonized cell may interact with C3b and other FP binding molecules on a different cell. FP‐driven agglutination of erythrocytes was associated with large non‐physiologically FP oligomers,^70^ but the contribution from the naturally occurring FP oligomers to agglutination of C3b opsonized bacteria is unknown. There is now one established example of such a cell‐bridging function of FP. The molecule may bridge C3b opsonized bacteria with host innate lymphoid cells presenting the NKp46 receptor, an interaction shown to be required for survival in an animal model of *Neisseria meningitides* infection.^71^ Lack of this cell‐bridging function may be an important element in the phenotypes of FP deficiencies in addition to compromised convertase formation and stabilization due to weaker binding to C3b on opsonized pathogens. Interestingly, administration of recombinant FP with a high content of FP tetramers and higher oligomers was protective in mouse models of infection with *N. menigitidis* and *S. pneumonia*.^72^ Bridging of bacteria and innate lymphoid cells by larger FP oligomers may have contributed significantly to the beneficial effects of recombinant FP administration.

Whereas multiple parallel alphafold2 predictions for the tick evasion proteins are all very similar, predictions for the NKp46‐FP interactions are less convincing, but all place the junction between the two Ig domains of NKp46 in contact with the C3b binding site on TSR5. If true, NKp46 will compete for interaction of C3b‐containing complexes with oligomeric FP. Such a mutual exclusive interaction mode is in very good agreement with the proposed role of FP trimers and tetramers in bridging of C3b opsonized cells with NKp46 presenting immune cells outlined in (Figure 3G). FP binds to multiple types of NKp46‐expressing cells independent of C3b.^71^ Hence, NK cells may circulate with FP prebound to NKp46 and in this way be ready to sense C3b opsonized pathogens. Clearly, further mechanistic investigations are required to understand the full implications of the NKp46‐FP interaction that constitutes the only additional host protein interaction FP is known to engage in besides the classical function as binding partner for C3b and FB. If the receptor and FP indeed compete for the same binding site, it may be challenging to selectively eliminate C3b or NKp46 binding by targeting FP.

## 11 CONCLUDING REMARKS

The recent structures of FP monomers have dramatically improved the understanding of how the FP monomers interact to form the very stable oligomers and how mutations in the FP encoding gene lead to functional and structural defects at the protein level. Structural studies of the oligomers have also demonstrated that these are surprisingly rigid and suggested how their highly extended architecture may allow FP to bridge multiple cells presenting C3b or other FP binding molecules on their surface. The next major accomplishment in terms of structural biology may well be in situ studies of FP bound to a C3b opsonized activator at a resolution approaching that of negative stain EM. Such studies would hopefully clarify whether the fascinating extended structure of the oligomers observed in solution is maintained in activator‐bound FP.

## FUNDING INFORMATION

Our work on properdin was supported by the Lundbeck Foundation (BRAINSTRUC, grant no. R155‐2015‐2666) and the Danish Foundation for Independent Research (grant no 4181–00137).

## CONFLICT OF INTEREST

The authors declare that there are no competing interest.

## Data Availability

The data that support the findings of this study are openly available in the protein data bank at https://www.rcsb.org/ or at the Small Angle Scattering Biological data bank https://www.sasbdb.org/ under the entries listed in Table 1.
